# Meta-Analytic Gene-Clustering Algorithm for Integrating Multi-Omics and Multi-Study Data

**DOI:** 10.3390/bioengineering11060587

**Published:** 2024-06-08

**Authors:** Ulrich Kemmo Tsafack, Kwang Woo Ahn, Anne E. Kwitek, Chien-Wei Lin

**Affiliations:** 1 Division of Biostatistics, Medical College of Wisconsin (MCW), Milwaukee, WI 53226, USA; ukemmo@mcw.edu (U.K.T.); chlin@mcw.edu (C.-W.L.); 2 Department of Physiology, Rat Genome Database, MCW, Milwaukee, WI 53226, USA; akwitek@mcw.edu

**Keywords:** gene-clustering algorithm, meta-analysis, multi-omics data, weighted correlation network analysis (WGCNA), fixed-effects model

## Abstract

Gene pathways and gene-regulatory networks are used to describe the causal relationship between genes, based on biological experiments. However, many genes are still to be studied to define novel pathways. To address this, a gene-clustering algorithm has been used to group correlated genes together, based on the similarity of their gene expression level. The existing methods cluster genes based on only one type of omics data, which ignores the information from other types. A large sample size is required to achieve an accurate clustering structure for thousands of genes, which can be challenging due to the cost of multi-omics data. Meta-analysis has been used to aggregate the data from multiple studies and improve the analysis results. We propose a computationally efficient meta-analytic gene-clustering algorithm that combines multi-omics datasets from multiple studies, using the fixed effects linear models and a modified weighted correlation network analysis framework. The simulation study shows that the proposed method outperforms existing single omic-based clustering approaches when multi-omics data and/or multiple studies are available. A real data example demonstrates that our meta-analytic method outperforms single-study based methods.

## 1. Introduction

One of the major aims of molecular biology is to decipher the biological processes involved in a particular organism through its gene expression profile. Gene pathways and gene-regulatory networks are constructed not only to explain the biological mechanisms in a given condition, but also to identify the group of genes involved in the condition. For instance, the Tumor Necrosis Factor (TNF) signaling pathway helps explain cell proliferation, differentiation, and the modulation of immune responses [1,2,3]. The genes in this pathway are involved in the activity of TNF which is considered as an anti-cancer agent. However, a large number of genes of some pathways and networks and their complexity make it difficult to interpret such high-dimensional gene expression data, leading to more noise and imprecision [4]. Moreover, many genes are still to be studied to define novel gene pathways and networks.

Ke and Yoshikuni [5] showed that the fast increase in genome databases publicly available and the computational identification of gene clusters have unveiled genes for enzymes with essential functions, as well as several of unknown metabolic pathways. In addition, clustering methods for gene expression data helps identify homology, which is relevant in vaccine development [4]. Another application of gene clustering is to identify modules of co-regulated genes and reveal subtypes of diseases in the context of precision medicine [4,6], where treatments are rather assigned based on the patient’s molecular signatures instead of the clinical disease stage.

Gene-clustering algorithms aim to group correlated genes based on some biomarkers such as RNA expression levels. Oyelade et al. [4] reviewed several clustering methods for gene expression data. Depending on the type of data, clustering can be done on the genes, the samples and/or the time variable. They evaluated the existing clustering methods for gene expression which include hierarchical methods, partitioning methods, model-based methods, and soft methods like Fuzzy clustering. However, those methods cluster genes based on only one type of omics data even though all the omics types are involved in the mechanism of the appearance of phenotype coded in the genes. Thus, they ignore information that is present in the other types of omics data. Also, the observed correlations between genes might differ from one type of omics data to another. Multi-omics clustering has a couple of advantages over single-omics clustering: multi-omics clustering can lessen the data noise effect; also, it integrates the information from all omics data and is able to reveal some important aspects present only in a few omics data [7]. The methods reviewed by Rappoport and Shamir [7] generally include:

- Early integration which consists of concatenating all the omics data to one dataset then applying a single-omics clustering;
- Late integration which consists of integrating the clusterings obtained after applying single-omics clustering to each omics;
- The similarity-based methods which consist of computing similarity among sample for each omics, and then integrate them;
- The dimension reduction-based methods;
- The correlation and covariance-based methods which include the canonical correlation analysis (CCA) and the partial least square (PLS).

However, these methods were developed specifically to cluster samples, but not genes. The current literature lacks clustering methods that utilize multi-omics data for clustering genes.

Clustering thousands of genes requires a large sample size to achieve an accurate clustering structure. However, it is costly to collect a large number of samples with multi-omics data. Investigators often find multiple independent studies that attempt to answer the same questions at different locations or under different settings. For instance, multi-omics data from several studies can be downloaded from large databases such as GEO and TCGA. For those scenarios, meta-analysis is intriguing in that it summarizes the results from various studies. In this paper, we propose an algorithm called Multi-omics Meta-Analytic Gene Clustering (MMAGC) that performs gene clustering based on gene–gene correlation matrix using multi-omics data and multiple studies. The rest of the paper is organized as follows: the proposed method is described in Section 2. Section 3 provides the results of the simulations carried out. Section 4 presents real data application and discussion is provided in Section 5.

## 2. Methods

A gene-based clustering aims to partition the genes into disjoint groups, called clusters, such that genes in the same cluster are highly correlated. The proposed meta-analytic clustering method consists of three sequential steps: firstly the gene-to-gene correlation is computed using multi-omics data for each study. Secondly, the within-study gene-to-gene correlation matrices are combined to obtain a meta-analyzed gene-to-gene correlation matrix. Finally, gene clustering is performed by applying a modified weighted correlation network analysis (WGCNA ) [8].

### 2.1. Step I. Computing the Gene-To-Gene Correlation for Each Study Using Multi-Omics Data

In this section, we propose three correlation measures to quantify the correlation between two genes using multi-omics data. Let *K* be the number of studies considered. We assume that all the studies investigate the same list of genes; let *G* be the total number of genes. Let $S_{k}$ be the number of samples in each omics datum in study k, k=1,...,K. Let *V* be the number of omics for each gene in each study. Then, each omics datum in study *k* consists of a $G\times S_{k}$ matrix, where the value at the entry (i,j) of the matrix represents the single-omics data of the *i*th gene in the *j*th sample.

**Figure 1:**
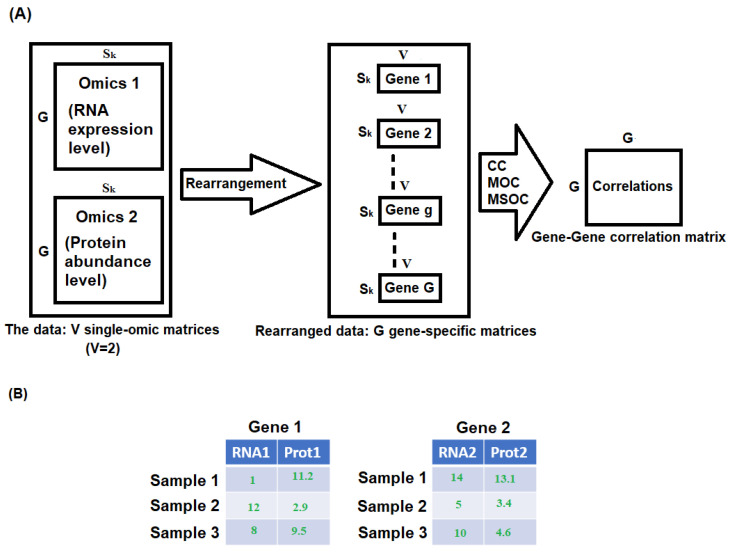
(**A**) A schematic example of obtaining the gene-to-gene correlation matrix from multi-omics data from study *k* via CC, MOC, and MSOC methods. Given study *k*, for gene g, g=1,...,G, we first construct a $S_{k}\times V$ gene-specific matrix, whose value at entry (i,j) represents the *j*th omics expression of gene *g* in the *i*th sample. (**B**) A toy example of two gene matrices with two omics data—RNA expression counts and Protein abundance levels.

We propose three ways of computing the correlation between two genes using multi-omics data: (i) canonical correlation (CC); (ii) correlation with the highest magnitude among all possible correlations between different type of omics data in two genes, which we name the maximum omics correlation (MOC); (iii) and correlation with the highest amplitude among the correlations between the same type of omics in two genes, which we name the maximum same omics correlation (MSOC). A schematic example is illustrated in Figure 1.

Using the toy example in Figure 1B, CC [9] between gene 1 and gene 2 is defined as the highest correlation between a linear combination of RNA1 and Prot1 and a linear combination between RNA2 and Prot2. The CC matrix for study *k* is the G×G matrix obtained by computing CC between all the pairs of genes from the genes considered in study *k*; and its entries are between 0 and 1.

Next, we define MOC between gene 1 and gene 2 as the strongest correlation out of the following 4 correlations: correlation between RNA1 and RNA2, RNA1 and Prot2, Prot1 and RNA2, Prot1 and Prot2. Its value is between -1 and 1. The MOC matrix for study *k* is the G×G matrix obtained by computing MOC between all the pairs of genes from the genes considered in study *k*.

We define MSOC between gene 1 and gene 2 as the strongest correlation out of the following 2 correlations: correlation between RNA1 and RNA2, and between Prot1 and Prot2. It ranges from −1 to 1. The MSOC matrix for study *k* is the G×G matrix obtained by computing MSOC between all the pairs of genes from the genes considered in study *k*. Because CC is nonnegative, we use the absolute value of MSOC and MOC when preparing the gene-to-gene correlation matrix for clustering genes.

### 2.2. Step II. Combining the Within-Study Multi-Omics Gene-To-Gene Correlations from Multiple Studies

In this section, we propose a method to combine gene-to-gene correlations obtained from different studies. Let $p^{*}=G\left(G-1\right)/2$ be the total number of distinct gene-to-gene correlations between the *G* genes. Let $r_{k}$ be the $p^{*}\times 1$ vector of estimated distinct gene-to-gene correlations in study *k* as described in Section 2.1, and ρ be the $p^{*}\times 1$ vector of the true distinct gene-to-gene correlations.

Hafdahl [10] proposed a fixed effects linear regression model to combine multiple Pearson correlation matrices for single-omics data. Inspired by Hafdahl [10], we consider the following fixed effects linear model:

(1) $$r_{k}=\rho +e_{k},\;\;k=1,\ldots ,K,$$

where $e_{k}$ is a $p^{*}\times 1$ vector error that follows the $p^{*}$-variate normal distribution with mean 0 and covariance matrix $T_{k}$. For single-omics data, the entries of $T_{k}$ can be estimated as follows:

(2) $$\hat{Cov(r_{ab,k},r_{cd,k})}=\frac{\left[\begin{array}{c} \frac{1}{2}r_{ab,k}r_{cd,k}\left(r_{ac,k}^{2}+r_{ad,k}^{2}+r_{bc,k}^{2}+r_{bd,k}^{2}\right)+r_{ac,k}r_{bd,k}+r_{ad,k}r_{bc,k}-\\ \left(r_{ab,k}r_{ac,k}r_{ad,k}+r_{ba,k}r_{bc,k}r_{bd,k}+r_{ca,k}r_{cb,k}r_{cd,k}+r_{da,k}r_{db,k}r_{dc,k}\right) \end{array}\right]}{S_{k}},$$

where a,b,c,d are genes, $r_{ab,k}$ is the estimated correlation between genes *a* and *b* in study *k* [10,11]. From Equation (2), we obtain

(3) $$\hat{Var(r_{ab,k})}=\frac{{\left(1-r_{ab,k}^{2}\right)}^{2}}{S_{k}}.$$

Note that Equation (2) is established for Pearson correlations. However, Muirhead and Waternaux [12] studied the asymptotic distributions of canonical correlations for non-normal distributions and showed that the asymptotic variance of the canonical correlation is as given in Equation (3) in both normal and non-normal cases. Intuitively, this was expected because the canonical correlation is a Pearson correlation of canonical variates. Since CC, MOC and MSOC are all Pearson correlations between variables, we use Equation (2) for their covariance estimation.

Using the generalized least squares, the estimate of ρ in Equation (1) is

$$\hat{\rho }={\left(\sum_{k=1}^{K}T_{k}^{-1}\right)}^{-1}\sum_{k=1}^{K}T_{k}^{-1}r_{k}.$$

It is computationally expensive to estimate the $p^{*}\times p^{*}$ matix $T_{k}$ for each study. Therefore, we consider estimating ρ taking $T_{k}$ as a diagonal matrix to reduce the computational cost.

### 2.3. Step III. Gene Clustering Using the Modified WGCNA

**Figure 2:**
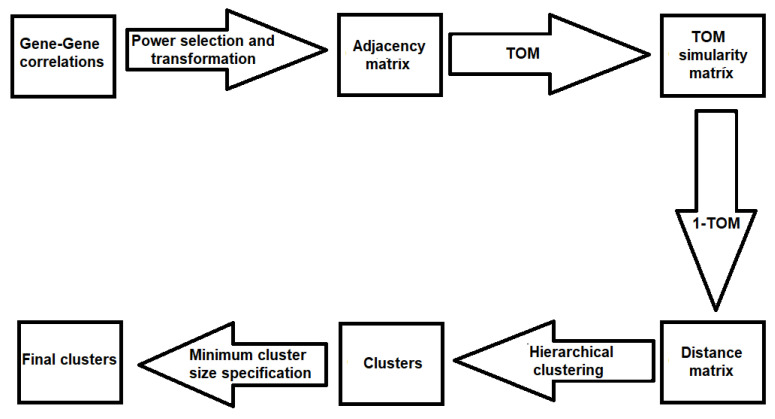
WGCNA flowchart summarized in 5 steps: power selection and transformation, TOM, 1-TOM, hierarchical clustering and minimum cluster size specification.

We propose a modified WGCNA to conduct gene clustering in this section. Figure 2 shows the flowchart of WGCNA [8]. WGCNA takes the gene-to-gene correlations as inputs and then the correlation matrix undergoes power transformation to suppress weak correlations. The adjacency matrix is obtained after applying the power transformation on the gene-to-gene correlation matrix. Then, the topological overlap measure (TOM) similarity matrix is computed to assess the similarity between genes based on the topology of the network. TOM values range between 0 and 1. A distance matrix is obtained from the TOM matrix by subtracting its values from 1. Then, the distance matrix is used for hierarchical clustering. The resulting clusters can be refined by specifying the minimum cluster size. We modify how to select the power in this WGCNA algorithm in Section 2.3.1.

#### 2.3.1. Power Transformation

In this section, we describe the existing and proposed power-selection approaches. The power function is defined as

$$f\left(x\right)=x^{m},$$

where *m* is an integer. The power function is applied to each entry of the gene-to-gene correlation matrix to suppress noise correlation between genes. It makes weak gene-to-gene correlations close to zero so that one can easily distinguish weak and strong correlations. Using a high value of *m* makes many correlations too close to zero. So it may cause strong correlations to vanish and thus reduce the expected number of connected genes called mean connectivity. Hence, the mean connectivity is a decreasing function of the power. Ideally, the power should be selected such that the mean connectivity does not decrease too much. The commonly used criterion is choosing the power that guarantees at least 60% adjusted $R^{2}$ goodness of fit while having a mean connectivity change rate of at most 1/3 [8]. In our simulation studies, we observed that this criterion tends to select very high power value, thus leading to mean connectivity close to 0. Therefore, we propose to select the power where the first local extremum of the rate of change of the mean connectivity change rate appears. This corresponds to where the rate of change (or slope or first derivative) of the mean connectivity significantly changes its concavity for the first time, which is the first inflection point of the mean connectivity change rate. As in Section 3, the proposed power-selection method improves clustering accuracy compared with the traditional approach.

After applying the chosen power to the canonical correlation matrix, we obtain a finer gene similarity matrix called adjacency matrix.

#### 2.3.2. Computation of the TOM Similarity Matrix

In this section, we describe how the topological overlap measure is computed. Using the adjacency matrix from Section 2.3.1, we obtain another similarity matrix based on the topology of the network. As Zhang and Horvath [13] demonstrated, similarity based on the network topology leads to more cohesive clusters than that from a scale free network. TOM is used to measure the agreement between the neighborhoods of two genes in a network. It is a normalized number between 0 and 1, of the number of neighbors that a pair of genes share [14]. Given the adjacency matrix $A=\left(a_{g_{1},g_{2}}\right),g_{1},g_{2}\in \left\{1,...,G\right\}$, TOM is defined as

$$TOM_{g_{1},g_{2}}=\left\{\begin{array}{ccc} \frac{l_{g_{1},g_{2}}+a_{g_{1},g_{2}}}{\min\left\{k_{g_{1}},k_{g_{2}}\right\}+1-a_{g_{1},g_{2}}}, & if & g_{1}\neq g_{2},\\ 1, & if & g_{1}=g_{2}, \end{array}\right.$$

where $l_{g_{1},g_{2}}=\sum_{g\neq g_{1},g_{2}}a_{g_{1},g}a_{g,g_{2}}$ and $k_{g_{1}}=\sum_{g\neq g_{1}}a_{g_{1},g}$.

Note that in an unweighted network, where the entries of the adjacency matrix are either zero or one, $l_{g_{1},g_{2}}$ is the number of shared neighbors of nodes $g_{1}$ and $g_{1}$ [15]. Yip and Horvath [14] showed that $TOM_{g_{1},g_{2}}$ is between 0 and 1 when the entries of *A* range from 0 to 1. The more (less) two genes have their neighbors connected, the closer to one (zero) their TOM value is.

#### 2.3.3. Hierarchical Clustering

We describe how hierarchical clustering is performed using the TOM matrix in this section. Since TOM is a similarity measure with value between zero and one, one minus the TOM measures the dissimilarity or the distance between two genes. The hierarchical clustering algorithm [16] is used with the distance matrix being one minus the TOM matrix. We choose the hierarchical clustering method because the number of clusters does not need to be specified in advance [16]. However, the minimum cluster size needs to be specified to prune the clusters output by the hierarchical clustering.

## 3. Simulation and Results

We conducted simulation studies to evaluate the performance of the proposed method. We assumed there are 5 studies with 50 genes partitioned into 10 clusters of size 5 each. Each gene had three types of omics. We considered the following four types of correlations:

- Correlation between the omics within the same gene: $\tau_{1}$;
- Correlation between the same type of omics of different genes in the same cluster: $\tau_{2}$;
- Correlation between different types of omics of different genes in the same cluster: $\tau_{3}$;
- Correlation between omics from different clusters: $\tau_{4}$.

Each three-omics dataset was generated using the multivariate normal distribution of mean 0 and the correlation matrix as the covariance matrix. In reality, the observed gene-to-gene correlation may not be the same in all the single-omics data. For instance, the correlation between 2 genes observed in RNA expression levels data might be different from that observed in the protein level data. To model this, we introduced the background correlation, $\tau_{5}$, and constructed perturbed clusters by changing the correlation between omics type 1 of genes 1, 2 and 3 and omics type 1 of all other genes to $\tau_{5}$. Then, only omics type 1 data information was perturbed. We allowed a proportion *p* of the clusters to have the error. We ran 100 iterations. Since there is no established order between the within-gene correlation and the correlation between the same type of omics of different genes of the same cluster, two scenarios were examined: when $\tau_{1}\geq \tau_{2}$ (scenario 1) and when $\tau_{1}\leq \tau_{2}$ (scenario 2). For each scenario, three cases were considered: when all five studies are independent and identically distributed (iid); when three studies are iid and two are iid with different values of *p*; and when none of the studies are iid with different values of $\left(\tau_{1},\tau_{2}\right)$. We examined three correlation matrix types to quantify gene-to-gene correlations: Canonical correlation (Meta_CC), MOC (Meta_MOC) and MSOC (Meta_MSOC).

When fitting fixed effects linear regression, estimating the covariance matrix of the error term is computationally costly. Thus, we also conducted simulations using the diagonal elements of $T_{k}$ only (Meta_CC_Diag, Meta_MOC_Diag, Meta_MSOC_Diag). Although this ignores correlation between error terms, it is computationally much more efficient. In addition, we compared the proposed methods with clustering based on a single-omics dataset as follows:

- The modified WGCNA where the first power, which maximizes or minimizes the rate of change of the mean connectivity change rate, was selected: WGCNA( 1);
- The commonly used WGCNA where the power selected is the one that guarantees at least 60% adjusted $R^{2}$ goodness of fit while having a mean connectivity change rate of at most 1/3: WGCNA(2);
- The *K*-means method [17] with the true number of clusters *c*: K-M(1). It was run 25 times where the *c* starting points were randomly chosen and the best result was picked, based on minimizing the within-cluster variance. In the real world, the true value of *c* is unknown, so this method is favored compared to the other single-omics methods we examined;
- The *K*-means method where the gap statistic [18] was used to choose the value of *c*: K-M(2). The value of *c* was chosen from 1 to the true value of *c* plus 10. This was also run 25 times and the best result was picked.

The adjusted Rand index (ARI) [19] between the true clusters and the predicted clusters was used to assess the performance of the methods. ARI measures the agreement between two clusterings. It has zero expected value in the case of random partition and it is bounded above by 1 in the case of perfect agreement between two partitions [19]. So, the closer to one the ARI is between the true and predicted clustering, the better it is.

### 3.1. Scenario 1: The Within-Gene Correlation ($\tau_{1}$) Is Greater or Equal to the Correlation between the Same Type of Omics of Different Genes of the Same Cluster ($\tau_{2}$)

Here, we explore the scenario where correlation between the omics of the same gene is greater or equal to that between the same type of omics of different genes of the same cluster. Three cases were examined in this scenario: when all the studies are independent and identically distributed (iid), when there is a mixture of 2 groups of iid studies, and when none of the studies are iid. The simulation was run with the sample size n=100.

#### 3.1.1. Case 1: All The Studies Are Independent and Identically Distributed (iid)

This case is illustrated with a slightly strong within-gene correlation $\tau_{1}=0.6$ and a moderate value of correlation between genes of the same cluster $\left(\tau_{2},\tau_{3}\right)=\left(0.4,0.3\right)$. Theoretically, the between-cluster correlation $\tau_{4}$ should be zero. But in practice, genes from different clusters may be very weakly correlated. To reflect this in simulation studies, we set $\tau_{4}=0.1$, the same value as the background correlation $\tau_{5}=0.1$. We set p=30%.

**Figure 3:**
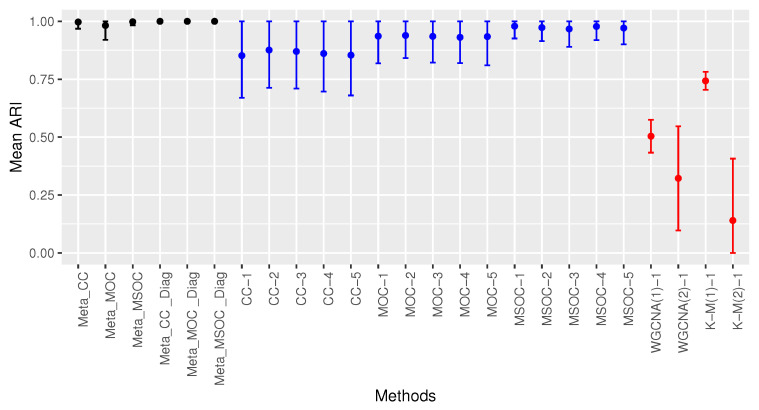
Comparing the clustering methods in Case 1 of Scenario 1. Multi-study multi-omics methods are drawn in black, single-study multi-omics methods are in blue and single-study single-omics methods are in red. The modified WGCNA outperforms the traditional WGCNA. Multi-omics single-study methods outperform single-omics single-study methods. Multi-omics multi-study methods outperform multi-omics single-study methods. The diagonal covariance matrix methods perform the best with perfect clustering.

Figure 3 shows the performance of the proposed methods as well as the multi-omics single-study methods based on the proposed methods. It also includes the performance of some single-omics single-study methods. The single-omics single-study methods were applied to the first omics type data of the first study only and the multi-omics single-study methods were applied to all the studies. For instance CC-1, CC-2,…, CC-5 are the CC method applied to the first, second,…, fifth study, respectively. The same goes for the MOC and MSOC methods.

Figure 3 presents the mean ARI of each method represented by the bold black dots. The implemented methods are presented in the horizontal axis. The vertical solid line represents the range of its mean ARI plus or minus its standard deviation with the lower bound being minimized by 0 and the upper bound being maximized by 1. Figure 3 shows that the multi-omics multi-study methods performs the best, followed by the multi-omics single-study methods. The best single-omics single-study method after K-M(1) is the modified WGCNA, WGCNA(1). This shows that the proposed method of selecting the power is superior to the traditional method. Among the multi-omics single-study methods, the MSOC performs the best, followed by the MOC. All the multi-omics multi-study methods with the diagonal correlation covariance matrix for the error terms in the fixed effects linear regression perform better than using the entire covariance matrix for the error terms. The former led to perfect clustering with the mean ARI being 1.

**Figure 4:**
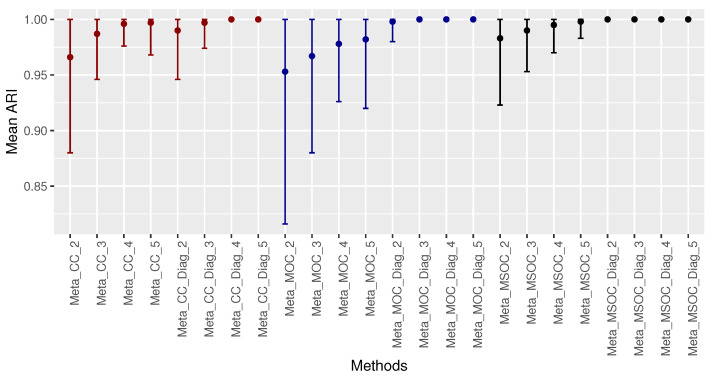
Comparing the multi-study clustering methods with respect to the number of studies, in the first case of scenario 1. The methods using the MSOC are drawn in black, those using the MOC are drawn in dark blue and those using the CC are drawn in dark red. The performance increases with the number of studies. The methods using the diagonal correlation covariance matrix outperform those using the whole matrix. Meta_MSOC_Diag is the best, followed by Meta_MOC_Diag.

Figure 4 shows the mean ARI of the multi-study methods with respect to the number of studies. The methods and number of studies are indicated in the horizontal axis. For instance, Meta_CC_2, Meta_CC_3,…, Meta_CC_5 are the multi-study CC method applied to the first 2, 3,…, 5 studies, respectively. The same goes for the Meta_MOC, Meta_MSOC, Meta_CC_Diag, Meta_MOC_Diag, and Meta_MSOC_Diag methods. No matter the number of studies, the methods using the diagonal correlation covariance matrix for the error terms in fixed effects linear models outperformed the corresponding method that used the whole correlation matrix. Also, the Meta_MSOC_Diag method started doing perfect clustering from 2 studies while Meta_MOC_Diag started doing perfect clustering from 3 studies and Meta_CC_Diag started doing it from 4 studies.

#### 3.1.2. Case 2: A Mixture of Two Groups of iid Studies with Different Values of *p*

This section reports the results we obtained in the case of a mixture of two groups of iid studies with different values of *p*. To illustrate this case, we considered that the first 3 studies were iid studies with the same parameters as in the previous case $\left(\tau_{1},\tau_{2},\tau_{3},\tau_{4},\tau_{5},p\right)=\left(0.6,0.4,0.3,0.1,0.1,0.3\right)$ and the last 2 studies were iid with the same parameters as in the previous case but with higher proportion of clusters with errors p=50%.

**Figure 5:**
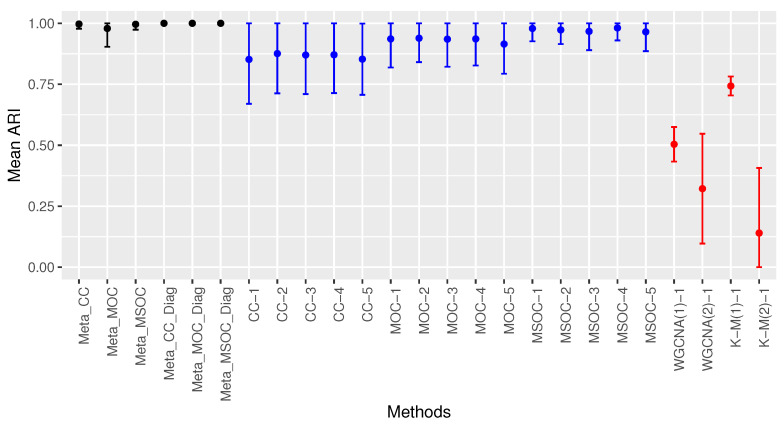
Comparing the clustering methods in Case 2 of Scenario 1. Multi-study multi-omics methods are drawn in black, single-study multi-omics methods are in blue and single-study single-omics methods are in red. The modified WGCNA outperforms the traditional WGCNA. Multi-omics single-study methods outperform single-omics single-study methods. Multi-omics multi-study methods outperform multi-omics single-study methods. The diagonal covariance matrix methods perform the best with perfect clustering.

Figure 5 shows the mean ARI of all the methods considered. The results were not significantly different from those of Case 1. The multi-study methods with a diagonal correlation covariance matrix performed the best with perfect clustering. Multi-omics single-study methods outperformed single-omics single-study methods and the proposed modified WGCNA outperformed the traditional WGCNA. Among the multi-omics single-study methods, the one using MSOC performed the best, followed by that using MOC.

#### 3.1.3. Case 3: None of the Studies Are iid with Different Values of $\left(\tau_{1},\tau_{2}\right)$

In this section, we considered that all the studies were independent, but not identically distributed. We considered the first study having the same parameters as those of Case 1, that is, $\left(\tau_{1},\tau_{2},\tau_{3},\tau_{4},\tau_{5},p\right)=\left(0.6,0.4,0.3,0.1,0.1,0.3\right)$. For the other four studies, we modified the values of $\tau_{1}$ and $\tau_{2}$ by ±0.1 while keeping the other parameters unchanged. More specifically, we set for study 2 $\left(\tau_{1},\tau_{2},\tau_{3},\tau_{4},\tau_{5},p\right)=\left(0.7,0.5,0.3,0.1,0.1,0.3\right)$; for study 3 $\left(\tau_{1},\tau_{2},\tau_{3},\tau_{4},\tau_{5},p\right)=\left(0.5,0.4,0.3,0.1,0.1,0.3\right)$; for study 4 $\left(\tau_{1},\tau_{2},\tau_{3},\tau_{4},\tau_{5},p\right)=\left(0.6,0.5,0.3,0.1,0.1,0.3\right)$; and for study 5 $\left(\tau_{1},\tau_{2},\tau_{3},\tau_{4},\tau_{5},p\right)=\left(0.5,0.5,0.3,0.1,0.1,0.3\right)$.

**Figure 6:**
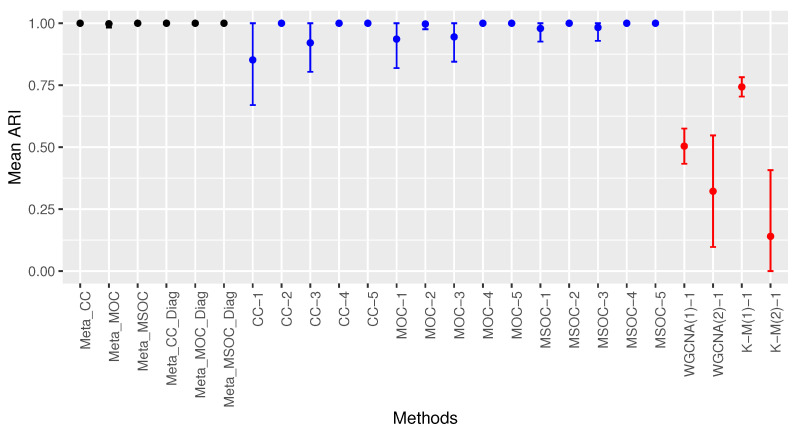
Comparing the clustering methods in Case 3 of Scenario 1. Multi-study multi-omics methods are drawn in black, single-study multi-omics methods are in blue and single-study single-omics methods are in red. The modified WGCNA outperforms the traditional WGCNA. Multi-omics single-study methods outperform single-omics single-study methods. Multi-omics multi-study methods outperform multi-omics single-study methods. The diagonal covariance matrix methods do perfect clustering.

Figure 6 shows the mean ARI of all the methods considered. The multi-study methods with a diagonal correlation covariance matrix for the error terms in a fixed effects linear model did perfect clustering with some methods that used the whole covariance matrix for the error terms (Meta_CC and Meta_MSOC). Also, the multi-omics single studies did perfect clustering in studies 2, 4 and 5 when $\tau_{2}$ was 0.5. In studies 1 and 3 where the value of $\tau_{2}$ was 0.4, the MSOC method performed the best, followed by the MOC method. Note that with a lower value of $\tau_{2}$ (0.4), the multi-omics single-study methods did not perform as well as they did when the value of $\tau_{2}$ is higher (0.5). With the same value of $\tau_{2}$ (0.4) as in studies 1 and 3, those methods performed better in study 3 where the value of $\tau_{1}$ was lower (0.5).

### 3.2. Scenario 2: The Within-Gene Correlation ($\tau_{1}$) Is Less than or Equal to the Correlation between the Same Type of Omics of Different Genes of the Same Cluster ($\tau_{2}$)

In this section, we explored the scenario where the correlation between the omics of the same gene is less than or equal to that between the same type of omics of different genes of the same cluster. The same cases of Scenario 1 were examined in this scenario. The same parameters were used except that the previous values of $\tau_{1}$ and $\tau_{2}$ were interchanged. This made the signal too strong—so strong that all the multi-omics single-study methods did perfect clustering. That observation goes along with that of the third case of Scenario 1 where the performance of those methods increased with $\tau_{2}$ but decreased with $\tau_{1}$. Therefore, a smaller sample size n=30 was considered in this scenario. For the first case, the parameter values were $\left(\tau_{1},\tau_{2},\tau_{3},\tau_{4},\tau_{5},p\right)=\left(0.4,0.6,0.3,0.1,0.1,0.3\right)$. In the second case, we considered the instance where the first 3 studies are iid studies with the same parameters as in the previous case $\left(\tau_{1},\tau_{2},\tau_{3},\tau_{4},\tau_{5},p\right)=\left(0.4,0.6,0.3,0.1,0.1,0.3\right)$ and the last 2 studies are iid with the same parameters as in the previous case but with a higher proportion of clusters with errors p=50%. And in the third case, we considered the first study to have the same parameters as those of Case 1 of this scenario, that is, $\left(\tau_{1},\tau_{2},\tau_{3},\tau_{4},\tau_{5},p\right)=\left(0.4,0.6,0.3,0.1,0.1,0.3\right)$. For the other four studies, we modified the values of $\tau_{1}$ and $\tau_{2}$ by ±0.1 while keeping the other parameters unchanged. More specifically, we set for study 2 $\left(\tau_{1},\tau_{2},\tau_{3},\tau_{4},\tau_{5},p\right)=\left(0.5,0.7,0.3,0.1,0.1,0.3\right)$; for study 3 $\left(\tau_{1},\tau_{2},\tau_{3},\tau_{4},\tau_{5},p\right)=\left(0.4,0.5,0.3,0.1,0.1,0.3\right)$; for study 4 $\left(\tau_{1},\tau_{2},\tau_{3},\tau_{4},\tau_{5},p\right)=\left(0.5,0.6,0.3,0.1,0.1,0.3\right)$; and for study 5 $\left(\tau_{1},\tau_{2},\tau_{3},\tau_{4},\tau_{5},p\right)=\left(0.5,0.5,0.3,0.1,0.1,0.3\right)$. The results were similar to those of scenario 1 and thus not included in the article.

### 3.3. Low Signal with Small Sample Size

**Figure 7:**
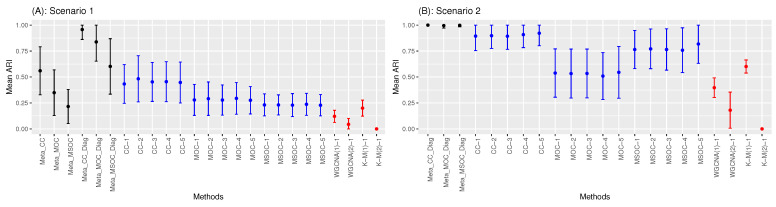
Comparing the clustering methods when the intrinsic signal is weak in Scenario 1 (**A**) and Scenario 2 (**B**). Multi-study multi-omics methods are drawn in black, single-study multi-omics methods are in blue and single-study single-omics methods are in red. CC outperforms MOC and MSOC.

All the variants of the proposed method did perfect clustering when the sample size was large enough. Their performance differed when the number of samples per study was small. The cases shown above were in the context of a strong intrinsic signal, that is, when the within-cluster correlation $\left(\tau_{1},\tau_{2},\tau_{3}\right)$ is not close to the between-cluster correlation $\tau_{4}$. However, the following simulations showed that the CC method outperformed MOC and MSOC in the context of weak signal and small sample size. That is, the signal is weak not because the sample size is limited but because $\left(\tau_{1},\tau_{2},\tau_{3}\right)$ is close to r4. This phenomenon is illustrated in Figure 7, which shows the mean ARI of all the methods in Scenario 1 (Figure 7A) and Scenario 2 (Figure 7B), with the sample size n=100. Figure 7A has the parameters $\left(\tau_{1},\tau_{2},\tau_{3},\tau_{4},\tau_{5},p\right)=\left(0.4,0.3,0.3,0.2,0.1,0.3\right)$ (Scenario 1) and Figure 7B has the parameters $\left(\tau_{1},\tau_{2},\tau_{3},\tau_{4},\tau_{5},p\right)=\left(0.3,0.4,0.3,0.2,0.1,0.3\right)$ (Scenario 2). In both scenarios, the CC methods were the best among the multi-study methods as well as single-study ones. However, the MSOC methods became the best when increasing the within-cluster correlation while keeping the between-cluster correlation and the sample size the same.

**Figure 8:**
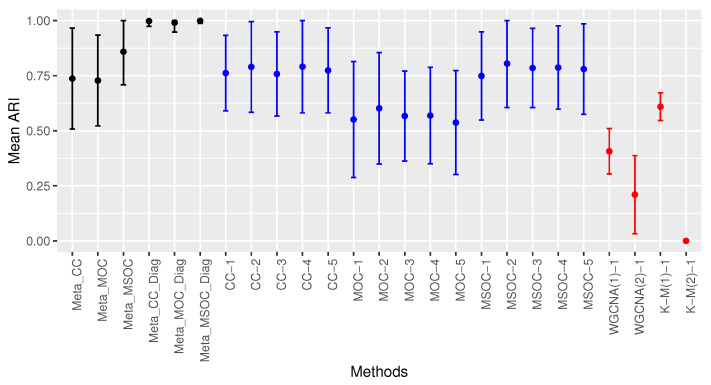
Comparing the clustering methods after a slight increase of within-cluster correlation from a weak intrinsic signal. Multi-study multi-omics methods are drawn in black, single-study multi-omics methods are in blue and single-study single-omics methods are in red. MSOC starts outperforming the CC and MOC.

This can be seen in Figure 8 which has $\left(\tau_{1},\tau_{2},\tau_{3},\tau_{4},\tau_{5},p\right)=\left(0.4,0.4,0.3,0.2,0.1,0.3\right)$. This involves increasing $\tau_{2}$ by a unit in Scenario 1 or increasing $\tau_{1}$ by a unit in Scenario 2.

Even though the MSOC single-study method and the CC one had similar performance, the MSOC multi-study methods outperformed the CC ones. It is worth noting that, in the cases illustrated in Figure 7 and Figure 8:

- The diagonal correlation matrix methods still outperform the whole matrix ones;
- The multi-study methods still improve the performance of the related single-study methods;
- The modified WGCNA methods still outperform the traditional one.

## 4. Real Data Analysis

In this section, we analyze human breast cancer data downloaded from the TCGA website, project TCGA-BRCA. The R package TCGAbiolinks was used to download two types of omics data: the RNA sequencing (RNA-seq) count data and the DNA methylation Beta-value data from the “Illumina Human Methylation 27” platform. The data cleaning and pre-processing include:

- Removing the genes with missing values;
- Matching the ensemble gene ID version to the HUGO gene symbol in the RNA dataset;
- Taking the average of duplicated samples if the average correlation of duplicates is higher than the average correlation of non-duplicate samples; otherwise, randomly selecting one of the duplicates;
- Taking the gene symbol with the highest coefficient of variation among duplicated gene symbols;
- Matching the probe ID to the HUGO gene symbol in the methylation dataset using the Illumina annotation file "HM27.hg38.manifest.gencode.v36.tsv" (downloaded from https://zwdzwd.github.io/InfiniumAnnotation#human, accessed date 30 May 2024);
- Taking the first listed gene symbol when the probe ID was associated with several genes;
- Taking average of methylation beta values across all CpG sites within the same gene;
- Normalization by applying the variance stabilizing transformation (implemented by vst function in DESeq2 R package) to the RNA-seq count data;
- Keeping the samples that are common to both datasets and getting rid of the genes with zero expression level in all those samples.

After pre-processing, each omics data had 313 samples in common and 123 genes overlapped.

**Figure 9:**
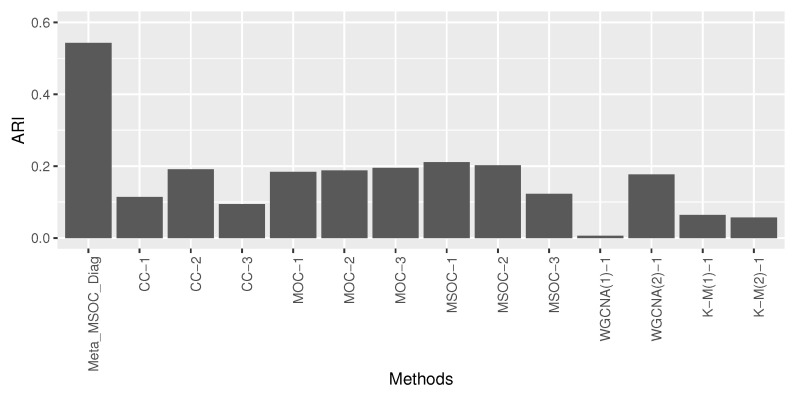
Barplot of the adjusted Rand index (ARI) between the clustering from each method using the subsampled dataset and the clustering using entire dataset. The meta-analytic MSOC method outperforms all single-study methods.

Since we did not know the ground truth of the gene clusters, we considered the clusters constructed by the entire dataset as the ground truth, with the MSOC method because it was the best among most simulation settings. Then, the entire study were randomly split into 3 studies with 99, 98 and 116 samples and treated as studies 1, 2 and 3, respectively. The performance of the MSOC meta-analytic method was compared with the other single-study methods, as we examined in Section 3. The results are presented in Figure 9 which shows the ARIs that compared the clustering result from each method to the ground truth obtained using the entire dataset with the MSOC method. The ARI of the Meta_MSOC_Diag method is clearly the highest among others, which shows that our proposed meta-analytic method outperforms the other single-study methods. Due to the relatively smaller sample size, we skipped the Meta_MSOC method in this example.

## 5. Discussion

Gene-clustering algorithms are widely used to identify unknown pathways. Multi-omics data allows the use of important information specific to each single-omics data for a better clustering result. Meta-analytic clustering is cost-effective because it integrates data from several existing studies for a robust result. We have proposed gene-clustering methods that integrate multi-omics data from multiple studies, using the fixed effects linear model and the modified WGCNA approach. The simulation studies show that the proposed methods perform the best when the diagonal correlation covariance matrix is used instead of the whole covariance matrix for the error terms in the fixed effects linear model. We suspect that the sample size n=100 is small to accurately estimate the covariance and thus results in large uncertainty in estimating parameters for the sample size. Thus, using the diagonal elements of the covariance matrix for the error terms performed better. In addition, with a large number of genes, the correlation covariance matrix for the error terms becomes not only computationally expensive, but also so memory consuming that the implementation of the method using a whole correlation covariance matrix can stop for lack of memory. When the within-cluster correlations are not close to the between-cluster correlation, the proposed method performs the best with the MSOC. However, when the within-cluster correlations are close to the between-cluster correlation, the proposed method achieves its best performance with the CC. The simulation studies also show that the proposed modified WGCNA outperforms the traditional WGCNA, and the multi-study methods outperform the single-study-based methods.

**Figure 10:**
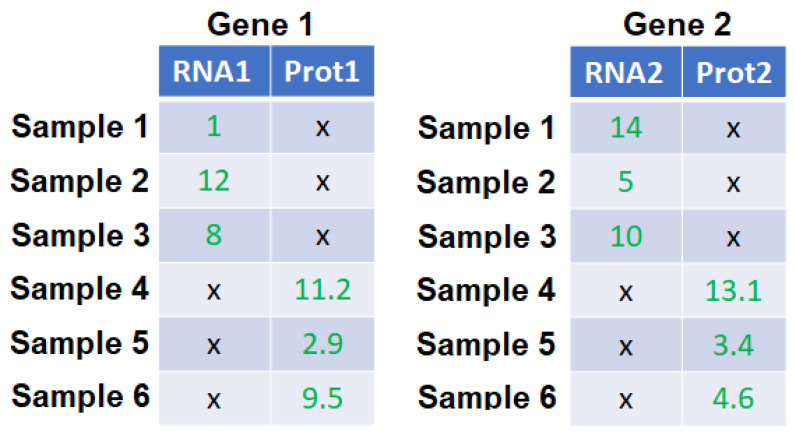
A toy example of two gene-specific matrices with two omics data—RNA expression counts and protein abundance levels; with missing values.

We assumed that the number of omics associated with a gene remains constant for all the genes. However, one can easily adapt the proposed method to accommodate varying numbers of omics associated with a gene across different studies. The same goes for the number of genes associated with each omics. Some studies have multi-omics data where the samples for each single-omics datum do not intersect. That leads to gene-specific matrices with missing values as illustrated by the toy example in Figure 10. Each gene has two omics data, the RNA expression counts and the protein abundance levels. However, RNA data are solely for samples 1, 2 and 3 and protein data are solely for samples 4, 5 and 6. In this situation, the MSOC can still be computed even though the CC and the MOC cannot. Therefore, the MSOC method is recommended for this kind of data.

The gene clusters identified with our method are based on statistical correlations. Thus, it does not reveal a causal relationship between genes. Further investigations should be conducted to understand the biological meaning of each gene cluster. In a simulation study, we did not consider some realistic scenarios, for instance, when within-gene correlations are different from different genes and when the between-gene correlations are different from the gene-to-gene ones. We anticipate this would require extensive simulation studies in the future. In practice, investigators may want to integrate the known correlation structure for certain genes when forming clusters. One potential approach is utilizing a weighted average of the established correlation and the observed correlation, either at the omics level or the gene level, within each study. The development of such a method warrants further investigation in the future.

Our software tool is implemented in R and is publicly available at https://github.com/ketsul14/MMAGC.git (accessed on 13 May 2024).

## Data Availability

Publicly available datasets were analyzed in this study. This data can be found here: https://www.cancer.gov/ccg/research/genome-sequencing/tcga (accessed on 13 May 2024).
